# How Do Alternative Protein Resources Affect the Intestine Morphology and Microbiota of Atlantic Salmon?

**DOI:** 10.3390/ani13121922

**Published:** 2023-06-08

**Authors:** Lucia Aidos, Giorgio Mirra, Margherita Pallaoro, Valentina Rafaela Herrera Millar, Giuseppe Radaelli, Chiara Bazzocchi, Silvia Clotilde Modina, Alessia Di Giancamillo

**Affiliations:** 1 Department of Veterinary Medicine and Animal Science, University of Milan, 26900 Lodi, Italy; lucia.aidos@unimi.it (L.A.); margherita.pallaoro@unimi.it (M.P.); chiara.bazzocchi@unimi.it (C.B.); silvia.modina@unimi.it (S.C.M.); 2 Department of Comparative Biomedicine and Food Science, University of Padua, 35122 Padova, Italy; giorgio.mirra@studenti.unimi.it (G.M.); giuseppe.radaelli@unipd.it (G.R.); 3 Department of Biomedical Sciences for Health, University of Milan, 20133 Milan, Italy; valentina.herrera@unimi.it

**Keywords:** Atlantic salmon, fishmeal replacement, intestine health, intestine morphology microbiota

## Abstract

**Simple Summary:**

The Atlantic salmon industry is expected to grow globally by 2–3% by the year 2030. The main protein source used in salmon feeds is fishmeal, which is produced from wild-caught marine fish, whose natural stocks are at risk of depletion. It is of major importance, therefore, to select alternative feed ingredients that present an adequate protein profile, guarantee a good health status and growth performance of Atlantic salmon, and at the same time are environmentally sustainable.

**Abstract:**

The availability and cost of fishmeal constitute a bottleneck in Atlantic salmon production expansion. Fishmeal is produced from wild fish species and constitutes the major feed ingredient in carnivorous species such as the Atlantic salmon. These natural stocks are at risk of depletion and it is therefore of major importance to find alternative protein sources that meet the nutritional requirements of the Atlantic salmon, without compromising the animals’ health. Terrestrial animal by-products have been used in aquaculture feed, but their use is limited by the lack of several essential amino acids and consumer acceptance. In the case of plant ingredients, it is necessary to take into account both their concentration and the extraction methodologies, since, if not dosed correctly, they can cause macro- and microscopic alterations of the structure of the gastrointestinal tract and can also negatively modulate the microbiota composition. These alterations may compromise the digestive functions, growth of the animal, and, ultimately, its well-being. An updated revision of alternative protein sources is provided, with the respective impact on the intestine health in terms of both morphology and microbiota composition. Such information may constitute the premise for the choice and development of Atlantic salmon feeds that guarantee fish health and growth performance without having a significant impact on the surrounding environment, both in terms of depletion of the fish’s natural stocks and in terms of pressure on the terrestrial agriculture. The sustainability of aquaculture should be a priority when choosing next-generation ingredients.

## 1. The Salmonid’s Gastrointestinal Tract (GIT)

Due to a large number of species, it is difficult to settle on a general nomenclature for the digestive system anatomy in teleost fishes. It has been suggested that there is a broad division of the anatomy into the head-intestine, fore-intestine, also named anterior intestine, mid-intestine (MI), and distal-intestine (DI), even if the limits between these regions are difficult to assess. This anatomical nomenclature has been used by Løkka et al. [1] in a broad study on the intestinal morphology of the wild Atlantic salmon, and the same nomenclature will be used in this review. The following GIT description corresponds to a healthy animal.

The teleost’s gastrointestinal tract (GIT) varies in terms of intestinal length, level of looping, number of pyloric caeca, and the presence or absence of a proper stomach [2]. The head-intestine is composed of two elements, the oral or buccal cavity and the gill cavity, also named branchial or pharyngeal, which play a role in food transport and respiration. The anterior intestine includes the esophagus and the stomach, the latter is well-developed in carnivore fish, such as the Atlantic salmon. The MI is further divided into a first (with openings to the pyloric caeca, PC) and a second segment, in which the latter is often addressed as the correspondent mammalian ileum. The DI is very short and it is proposed as the equivalent mammalian colon and anus [3]. In this review, the head- and the anterior intestine are not considered, as the available literature does not refer to effects on these tracts that can be related to feed. For this reason, the description starts with the MI and then with DI.

### 1.1. Macroscopic Aspects of the Intestine

#### 1.1.1. Mid Intestine (MI)

The MI is the longest portion of the intestine and the luminal surface is remarkably folded, with prominent longitudinal major or primary mucosal folds, which are further subdivided into a series of irregular and well-circumscribed folds called minor or secondary folds. In Atlantic salmon, the first segment of the MI likely is the main site of nutrient absorption of the GIT and the folds increase the absorptive area, similar to what happens with the villi in the mammalian small intestine [4]. However, if in teleosts the folds of the first segment of the MI are longitudinal or with a branching, net-like pattern, in Atlantic salmon these appear longitudinally arranged, but the explanation for this has not been stated [1]. Pyloric caeca are present in the first segment of the MI and consist of blind extensions of the intestine that present small folds with a longitudinal orientation to the length of the caeca. The functions of the pyloric caeca include the secretion of digestive enzymes and the absorption of nutrients [5]. In Atlantic salmon, pyloric caeca account for 87% of the total intestinal length and 64% of the total post-gastric intestinal mass [6]. The second segment of the MI is characterized by macroscopically visible circular folds and it shows a higher diameter when compared to the first segment of the MI. Although the function of the circular folds is not fully known, a defecatory function has been hypothesized by Burnstock [7]. In Atlantic salmon, the second segment of the MI seems to have an important role in the antigenic uptake and it is immunologically more active than the other segments of the GIT, as reviewed by Bjørgen et al. [3]. Many authors showed that the wall of the second segment of the MI is thinner than in the first segment [1]. The thin wall and the broad folding of this region indicate that few mechanical processes take place.

#### 1.1.2. Distal Intestine (DI)

The DI is the shortest region of the intestine and its beginning is usually marked by an increase in diameter. The luminal surface presents small folds without an evident orientation, similar to the first segment of the MI [2,4]. Moreover, the wall of the DI in teleosts presents a thicker muscular layer than the other intestinal regions. This fact was confirmed in Atlantic salmon post-smolts, but not in spawning individuals, but the authors did not present an explanation for this fact [1]. The distal intestine has an important role in osmoregulation [8] and it is also a major site of absorption of intact proteins in all teleost fishes [9].

### 1.2. Microscopic Aspects of the Intestine: Mid and Distal Intestine (MI and DI)

**Figure 1:**
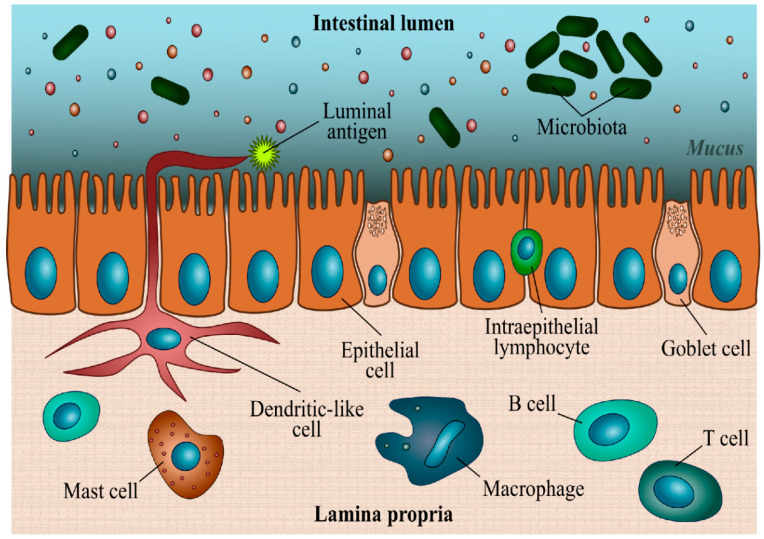
Intestinal mucosa in Atlantic salmon: it is possible to observe goblet cells and intra-epithelial lymphocytes in-between the epithelial cells. The lamina propria presents multiple groups of immune cells, such as dendritic-like cells, mast cells, macrophages, and B and T cells.

A description of the histology of all segments of the intestine of Atlantic salmon has been explained extensively elsewhere [1]. Therefore, only a summary of the intestine micro-anatomy is provided here. In vertebrates, the intestinal wall is composed of four layers: the innermost layer is the tunica mucosa, underneath there is the submucosa, followed by the muscularis, and the outer layer is the serosa [10]. Even if in teleosts the same nomenclature is used, there is some discussion on which tissues belong to the mucosal layer and which ones belong to the submucosa. Figure 1 shows the heterogeneous cell populations that can be found in the mucosa.

The inner mucosal layer is made of one layer of cells, and an underlying lamina propria of connective tissue. The epithelium is composed of a monolayer of four cell types: enterocytes, goblet, endocrine, and immune cells [11,12,13]. The epithelium has an important role in nutrient transport, osmoregulation, and protection from environmental stressors [14,15]. Enterocytes are columnar cells that present a brush border, which is a microvilli-covered surface, and are responsible for the absorption of nutrients. Tight junctions are structures that form contact points between enterocytes and are responsible for regulating the permeability of ions, (macro)molecules, and cells via the paracellular pathway. Goblet cells are mucus-producing cells that constitute the inner mucus layer. Endocrine cells respond to the presence or absence of food in the intestine by secreting peptide hormones. The intraepithelial lymphocytes are the first immune cell line in the intestine that is involved in defense actions (Figure 1). These lymphocytes, which are located between epithelial cells, present mobility and control the space between epithelial cells above the basement membrane [16]. Noteworthy, mammals present villi which are small, finger-like projections made up of cells that line the small intestine. In fish, these structures are completely absent throughout all the tracts. However, it is important to stress that some authors define the folds as villi [17] regarding the presence of crypt-like structures at the base, similar to those described in mammals.

As described by Bjørgen et al. [3] for all the intestinal segments, the lamina propria is located underneath the basal membrane below the epithelium and it is composed of connective tissue containing leukocytes. Precisely, in Atlantic salmon in the lamina propria, it is possible to find mast cells, macrophages, dendritic cells (antigen-presenting cells), B cells (IgM-positive cells), and T cells [18] (Figure 1). Mast cells play a crucial role in the innate and adaptive immune responses, acting against allergens, bacteria, toxins, parasites, neuropeptides, and stress by increasing epithelial secretion and peristalsis, and by releasing pro-inflammatory mediators. Intestine macrophages regulate the inflammatory reaction to bacteria and antigens that can pass the epithelium. Macrophages also have a role in the protection of the mucosa against harmful pathogens and in dead cell scavenging. Dendritic cells are responsible for the regulation of tolerance to food antigens and are also necessary for the optimal response to intestinal pathogens. B and T lymphocytes intervene in the specific responses of adaptive immunity, although in different modes: B cells express their antigen receptor on the cell surface and secrete them as an immunoglobulin (Ig) or antibody, while the T cell receptor is always bound to the cell surface [19].

The lamina propria lies on a thick layer of connective tissue, rich in collagen, called the stratum compactum: it is a typical characteristic of carnivorous fishes, and acts as a prevention of stomach distention during food ingestion. This layer is surrounded by the stratum granulosum, which presents a large number of mast cells. On the other hand, the submucosa presents a deeper, looser connective tissue than the lamina propria. The muscular layer, internally, presents a circular orientation of the muscle fibers, whereas externally the muscle fibers are disposed longitudinally.

As described by several authors [20,21], Paneth cells, which are highly specialized secretory epithelial cells that contain antimicrobial peptides that regulate the composition of the intestinal flora, seem to be missing in Atlantic salmon. In addition, as already mentioned, the crypts of Lieberkühn, which are intestinal glands, are absent [22]. Løkka et al. [1] found high proliferation rates of the columnar cells at the base of the primary and secondary folds. This suggests that the folds in teleosts have corresponding functions to the crypts in mammals, which are involved in cell proliferation, differentiation, and migration.

### 1.3. Microbiota

The intestine microbiota has a crucial role in all vertebrates [23], as commensal bacteria can influence important factors such as growth, development, homeostasis, and well-being [24]. In fish, as in the case of mammals, the intestine microbiota is composed of autochthonous or allochthonous bacteria [25]. The autochthonous bacteria are derived from the colonization of the host’s intestinal epithelial surface, while the allochthonous bacteria are transient [26].

Although the microbiota in fish has been studied since the beginning of the last century, information is still limited. Interactions between the host, diet, and environmental conditions contribute to altering the intestine microbiota [27], causing an imbalance in nutrient digestion and utilization [28,29], enzyme production [30], and also in immune status, which can decrease disease resistance [31,32]. It is therefore of great importance to evaluate the impact of food on the intestine microbiota.

## 2. Nutritional Needs of Salmon—“The Protein Problem”

Atlantic salmon requires the same nutrients (protein, amino acids, essential fatty acids—EFA, vitamins, and minerals) as other carnivorous fish species for normal growth, reproduction, and immune and metabolic functions, but the quantities and proportions are species-specific [33]. Precisely, dietary protein and amino acids supply are major factors influencing the productivity of farmed salmon [34]. Salmon fry shows better growth performances with high protein diets (~50%), while grow-out diets contain normally 42–48% protein. More than a general protein requirement, fish need essential amino acids, such as arginine, histidine, isoleucine, leucine, lysine, methionine, phenylalanine, threonine, tryptophan, and valine for regular growth. An unbalanced level of specific amino acids in the diet may result in mild or severe amino acid deficiency, mainly when fish are subjected to some environmental and physiological stress, even if there are certain essential amino acids such as leucine that, when present in high amounts in the diet, may be toxic [33]. When the fishmeal (FM) inclusion level in the diet of salmon is reduced and replaced with other protein sources, there may be an imbalance of essential amino acids. This is why we have “the protein problem”.

## 3. Fishmeal (FM) Dietary Replacement—Looking for Potential Candidates

In Atlantic salmon, the main used protein source ingredients are FM (anchovies, pilchards, mackerel, herring, and blue whiting), plant protein products (soybean meal, corn gluten meal, canola meal, pea meal), animal by-products meal (poultry by-product meal, meat meal, blood meal, hydrolyzed feather meal), and crustacean meal (krill, shrimp, crab). Among these, FM has been the preferred one because of its high protein content, excellent amino acid profile, high nutrient digestibility, and absence of antinutrients [35,36]. Atlantic salmon feed is usually composed of about 40–60% of FM. Indeed, in 2013, the aquaculture industry consumed about 56% of the world’s supplies of FM [37], and the demands are expected to increase as the industry grows. Taking into account that 80% of the world’s fish stocks are stated as fully or over-exploited [38], further growth in aquaculture production will be limited by FM availability and by its increasing price. Alternative feed ingredients will, therefore, be extremely necessary [39]. The possible options are vegetable proteins, by-products from fish and terrestrial animal processing industries, organisms from lower trophic levels, and industrially produced bio-protein, such as bacteria and algae [40].

However, it is important to bear in mind that the replacement of FM with terrestrial plant ingredients may induce changes and cause negative effects on the GIT. The use of plant ingredients is limited in carnivorous fish species due to the presence of starch and structural carbohydrates, and a wide range of anti-nutritional factors (ANF). [41,42,43,44]. Despite this, plant ingredients remain the most attractive protein sources due to their high protein content and availability. For instance, among plant protein sources, field peas are a good candidate in species such as Atlantic salmon [45], rainbow trout [46], and European sea bass [47], even if the protein content is lower than in other plant protein sources, such as oil seeds. Like soya, peas also contain several ANF but, as observed by Francis et al. [35], their content is lower when compared with other legumes.

The intestine’s health largely depends on the impact of the diet and can be assessed by evaluating histological parameters based on structural changes in the intestine itself. The most frequent alterations in the intestine include the reduction in the height of the folds, the simultaneous widening of the lamina propria, an increase in the presence of intra-epithelial lymphocytes, and a large amount of apical vacuolization of the columnar cells; all these histological signs reflect the reaction to the exposure to pathogens or stressors [48]. The impact of several protein sources on the structure of the DI and MI is summarized in Table 1 and will be further described in detail.

Diet can also influence the microbiota status, and many alternative plant-based proteins were investigated for their negative impact on the intestine [49]. The state of intestine health and the microbiota reflect the correct diet and the potential use that Atlantic salmon can make of it. Table 1 summarizes the studies that have focused on how different fishmeal substitutes have affected intestinal health, and Table 2 the effects on intestinal microbiota.

**Table 1:** Impact of alternative protein sources on the morpho-functionality of the mid and distal intestine in Atlantic salmon. Negative effect, −; positive effect, +; no effect, =; increase, ↑; decrease, ↓; * addition of probiotics. A higher number of these symbols, give an indication of severity degree of the respective impact.

Protein Source	Inclusion Level	Mucosa Folds	Mucosa Thickness	Mucosa Cells	Digestive/Absorption Capacity	Inflammatory Process	Overall Histology	References
Soybean Meal	10%				↓			[50]
	10%	−	−	−		↑		[51]
	15%	−−		−−	↓↓			[50]
	20%	−−−		−−−	↓↓↓			[50]
	20%	−−	−−	−−	↓	↑↑		[51]
	20%		−			↑		[52]
	20%	−	−					[53]
	20% *		+	+				[54]
	25%	−−−−		−−−−	↓↓↓↓			[50]
	30%		−		↓	↑	−	[55]
	30%	−		−				[49]
	32%	−	−		↓	↑↑	−	[56,57]
	35%	−	−	−		↑		[58]
	35%	−−−−		−−−−	↓↓↓↓↓			[50]
	40%	−−	−−	−		−		[57]
	50%	−	−	−		↑		[59]
HP48	17.8%	−	−	−				[60]
Whole Field Pea Pea Protein Concentrate	20%	=	=	=		=	=	[45]
	20%	=			↓		=	[61]
	35%	−	−	−	↓	↑	−	[43]
	44%	=	=	=			=	[62]
Dehulled Faba Faba Bean Concentrate	20%	=	=	=		=	=	[45]
	17%	−	−	−	↓	↑		[63]
	5–20%	=	=	=		↓	=	[64]
	35%	−−			↓↓	↑↑		[63]
	45%	=	=	−		↑	−	[64]
Plant Proteins	25%	=	=	=		↑		[65]
Poultry Meal	20%		−			=		[66]
	58%	=	=	=			=	[67]
Feather Meal	20%			−		=		[62]
Insect Meal	5–15%	=	=				=	[68]
	5–15%	=					=	[69]
	6.25–25%	=		=		=	+	[70]
	15%	−		−		↑	+	[71]
	25%	=	=	=			=	[17]
	85%	=	=	=			=	[71]
Microalgae								
*Nannochloropsis oceanica*	20%	=	=	=	↓	=	=	[72]
*Desmodesmus* sp.	20%	=	=	=			=	[73]
Yeasts								
*Candida utilis*	20%	+						[74]
	25%	=	=	=			=	[23]

**Table 2:** Effects of different protein sources on gut microbiota in Atlantic Salmon. +, presence.

Protein Source	Concentration	Intestinal Tract	Effects on Microbiota (MB)	Microbial Population Mostly Influenced	Enteritis	References
Soybean meal	5–10%	PI and DI mucosa	Yes	Presence of bacteria not normally associated with marine fish (*Escherichia* and *Propionibacterium*)		[75]
	20%	MI and DI mucosa	No	*Tenericutes* dominant Phylum *Proteobacteria*, *Firmicutes* and *Bacteroidetes.* Lactic acid bacteria increase	+	[52]
	20%	MI and DI digesta	Yes	Abundance of *Firmicutes* and *Bacteroides*; increase in *Proteobacteria*	+	[76]
	25%	MI and DI mucosa and digesta	Yes	Increase in *Enterococcus* spp., mostly *Enterococcus faecalis*		[41]
	30%	DI mucosa and digesta	Yes	*Proteobacteria*, *Firmicutes*, and *Bacteroidetes* Abundance of lactic acid bacteria	+	[49]
	30%	DI digesta	No	Dominated by lactic acid bacteria		[77]
	50%	DI digesta	Yes	*Shewanella* dominant bacteria. *Lactococcus, Aeromonas, Pseudomonas.* Lactic acid bacteria may modulate intestinal inflammation		[59]
Pea protein concentrate	20%	Allochthonous bacteria: Fecal samples Autochthonous Bacteria: PI and DI mucosa	Yes	*Corynebacteriaceaein* dominant in the allochthonous bacterial population and *Lactobacillaceaein* in the autochthonous		[62]
Poultry by-product meal	20%	Allochthonous bacteria: Fecal samples Autochthonous Bacteria: PI and DI mucosa	Yes	*Corynebacteriaceaein* dominant in the allochthonous bacterial population and *Lactobacillaceaein* in the autochthonous		[62]
Insect meal	60%	DI digesta and mucosa	yes	Increasing of *Actinomyces, Bacillus, Brevibacterium, Corynebacterium 1* and *Enterococcus*		[78]
Yeast *Cyberlindnera jadinii*	10%	MI and DI mucosa	yes	*Weissella* and *Leuconostoc* slightly increased. *Lactobacillus* decrease		[79]
	20%					

### 3.1. Soybean Meal (SBM) and Soy Protein Concentrate

The inclusion of soybean in feeds for animals, both land and aquatic, has been extensively studied [52,55,56,57,58,59,60,75,80,81,82,83,84]. Its low price and wide availability have made soybean a perfect candidate for the replacement of FM, and, as a whole, the literature has shown that the MI does not show significant alterations, regardless of the diet. On the other hand, the DI morphology was affected in fish fed the full-fat SBM, with an increased number of goblet cells in the epithelium, enterocytes with low or no absorptive vacuoles and shorter microvilli, and a thicker lamina propria. The condition of the inflammatory response of the mucosa of the DI after the inclusion of SBM is known as SBM-induced enteritis. The actual causative components and SBM mechanisms of action need further research. It is known that the etiology and development of SBM-induced enteritis are related to SBM inclusion levels and the commercial source used for the diet formulation. This can have a great impact on the severity of the disorder, mainly affecting the endocytosis process. Uran et al. [80] evidenced that the cytological endocytosis block is directly related to the disappearance of the supranuclear vacuoles, which can be considered as the most striking feature in the onset of enteritis.

The negative effects of SBM-induced enteritis do not appear to affect all fish species investigated [84]. In particular, carnivorous fish species such as Atlantic salmon suffer from intestinal inflammation after being fed high levels of plant components that contain anti-nutritional factors such as protease inhibitors, oligosaccharides, lectins, phytic acids, and saponins, contained in SMB [41,42,43,44,85].

Below is an overview of the literature from the last few years on intestine health and microbiota.

Intestine health—no effects: The effect of feeding conventional and genetically modified (GM) soya plant products could be an economic alternative and it was evaluated by Sanden et al. [86] in farmed Atlantic salmon parr by investigating intestinal indices (relative weight of each intestine portion), histology, and cell proliferation level. No changes were observed in the intestine of Atlantic salmon parr that could be associated with the inclusion of genetically-modified plant material in the feed.

Intestine health—negative effects: Baeverfjord and Krogdahl [57] performed one of the first studies on the changes in the intestine morphology in Atlantic salmon fed diets with FM or SBM. Fish were fed with an SBM diet (40% protein replacement) for 3 weeks and the DI presented the following negative features, typical of SBM-induced enteritis: (1) shorter primary and secondary mucosal folds; (2) loss of the supranuclear vacuolization of the absorptive cells of the intestinal epithelium; (3) increased amount of the connective tissue, resulting in an increase in the width of the central stroma in the mucosal foldings; (4) widening of the lamina propria; (5) great infiltration of inflammatory cells in the lamina propria. The authors observed, as well, that the inflammatory cells were composed of different cell populations: lymphocytes, macrophages and polymorphonuclear leucocytes, and diffuse immunoglobulin M (IgM). Moreover, the vasculature of the lamina propria appeared frequently dilated. This study constituted the first of a series of other studies where various inclusion levels and forms of SBM were investigated. In addition, Bakke-McKellep et al. [58] studied the impact of replacing FM with 35% SBM in the diet (both genetically modified and not genetically modified) and found that the DI was the only tissue that showed a significant negative variation in terms of lamina propria thickness, fold length, and in the number of the supranuclear vacuoles, regardless of the SBM type. With the same inclusion level of SBM, another study with Atlantic salmon showed an average reduction of 23% of the mucosal wet weight of the DI, as well as SBM-induced changes [55]. In the same study, the authors observed that SBM leads to a decrease in carrier-mediated transport and an increase in the permeability of the DI epithelium, with a diminished capacity of the DI to absorb nutrients. A recent study studied the effects of a soybean compound named HP48 which is made from solvent-extracted peeled soybeans [60]. The authors found that the HP48 diet causes negative morphological changes in the DI, such as the widening of the lamina propria and stratum granulosum, disrupted mucosal folds, and the loss of absorptive vacuoles.

Krogdahl [50] observed that in fish fed a diet containing FM as the only protein source, no relevant pathological changes were observed in the intestine; on the contrary, the authors observed that the inclusion of SBM from 10 to 35% of total protein resulted in a graded morphological response: an increase in the vacuolization of absorptive cells, height and width of mucosal folds, and cell infiltration of the submucosa and lamina propria.

The effect of the various inclusion levels of SBM in Atlantic salmon was assessed by Urán et al. [51], where fish were fed diets with 0%, 10%, and 20% SBM for 57 days. Already from day 5 in fish fed the 20% diet and from day 7 in fish fed the 10% diet, there were morphological changes. These consisted of a decrease in the supranuclear vacuoles, damaged and smaller mucosa folds, an increase in cell infiltration in the submucosa, an increase in the goblet cells, and a widening of the lamina propria and submucosa. These changes were greater in fish fed the 20% diet, and, for both diets, changes were more evident over time. Urán et al. [51] also found an evident reduction in microvilli after only 7 days of 20% SBM feeding, indicating a negative impact on the uptake capacity of the enterocytes of the DI. The already mentioned changes are in agreement with a more recent study by Booman et al. [52] where Atlantic salmon fed 20% SBM showed an increase in submucosa thickness and infiltrating cells.

Serious enteritis was described in the DI of the Atlantic salmon fed a diet with an SBM inclusion level of 32% by evaluating the following criteria: presence and size of supranuclear vacuoles, degree of widening of the lamina propria of simple folds, amount of connective tissue between the base folds and stratum compactum, and degree of thickening of the mucosal folds [56].

To investigate the role of the intestinal microbiota in SBM enteropathy, the effect of supplementing a high inclusion level of SBM (50%) with lactic acid bacteria (LAB) was assessed in Atlantic salmon [59]. As expected, in fish fed with non-supplemented SBM, the height of the mucosal folds, the presence of supranuclear vacuoles, the number of goblet cells, and the thickness of the lamina propria were all negatively affected. The supplementation of LAB to the SBM diet decreased the inflammation status, and all histological parameters of inflammation were reduced as well. The results of this study suggest that LAB may modulate intestinal inflammation in fish. Moreover, there is evidence that enteritis caused by the inclusion of SBM in the diet may increase fish susceptibility to bacterial disease [87]. A recent study on Atlantic salmon, aiming to compare several protein sources, found that fish that developed SBM-induced enteritis presented a lower expression of the muc2 mucin, the major protein component of mucus that lines the epithelia of the intestine, compromising the intestinal barrier status [88].

Thereby, it was clear that SBM at inclusion levels from 10 up to 50% led to an inflammation process in the DI of Atlantic salmon. The attention started to be focused on the causes of this SBM-induced enteritis and how to overcome these.

Some authors hypothesize that the causative agent of enteritis may presumably be one or more of the alcohol-soluble components of SBM [50]. In a study by Knudsen et al. [89], soybean molasses were separated into three subfractions, and all possible combinations of these subfractions were fed to Atlantic salmon. Fish fed the water phase subfraction displayed a normal morphology, but fish fed a combination of the butanol phase and the precipitate displayed the same morphological changes as fish that were fed soybean molasses. The authors concluded that the presence of soya saponins, or other components that show the same solubility pattern, is associated with the inflammatory reaction. The extrusion process, a technology that uses a high temperature and short time regime, together with intense mechanical shear [90] seems to inactivate the anti-nutritional factors present in SBM, but, still, research is ongoing to find other soya forms that do not produce negative effects such as enteritis.

Intestine health—positive effects: Catalán et al. [91] studied the effect of an alternative form of soybean on Atlantic salmon intestine health: the fermented SBM-based diet. The authors observed a boost in health and growth physiology in fish, promoted by intestinal lactic acid bacteria and an increase in the PI trans-cellular uptake of water. A similar diet could be tested on the DI, which seems to be the tract of the intestine most affected by SBM [41,50,55,56].

Krogdahl et al. [92] tested a new form of a non-genetically modified soybean, named Triple Null (TN), which is devoid of the Kunitz trypsin inhibitor, lectin, and allergens. The authors found that animals fed TN showed DI enteritis and a higher expression of pro-inflammatory genes, as well as two stress-related genes, when compared with the soy protein concentrate diet. The authors concluded that, most likely, the extrusion process used for producing feed seems sufficient to inactivate proteinaceous antinutrients, the removal of which does not affect the nutritional value of the feed for Atlantic salmon. The addition of probiotics to a 20% SBM diet has been evaluated on post-smolt Atlantic salmon [54]. The authors observed an increase in intestine supranuclear vacuoles, and reduction in the lamina propria width in animals fed the probiotics. It was concluded that, most probably, this addition may prevent enteritis.

Microbiota: Research on the effects of SBM on microbiota is still recent compared to that on the intestine morphology, and, for this reason, it is difficult to conclude the effective relationship between soybean and microbiota; however, Bakke-McKellep et al. [93] found that 25% SBM can alter the microbiota with an increase in the number and diversity of bacteria, especially Enterococcus spp., and mostly Enterococcus faecalis. The same authors found no relationship between microbiota alteration and SBM-induced enteritis. On the contrary, Reveco et al., [76] suggest that the modulation of the bacterial community with 20% of SBM could lead to SBM-induced enteritis; in this study, the authors report an abundance of Bacteridoites and Firmicutes, and also an increase in Proteobacteria. Similarly, Gajardo et al. [49] studied the inclusion of 15% soy protein concentrate, mixed with wheat gluten, and found an abundance of lactic acid bacteria in the mucosa-associated microbiota and alterations in the histomorphology, typical of SBM-induced enteritis; the phyla mainly represented in microbiota were Proteobacteria, Firmicutes, and Bacteroidetes. Although the phyla found were similar, Booman et al., 2018 [52] did not observe any relationship between 20% SBM and microbiota alteration in Atlantic salmon. In this study, the analysis showed that Tenericutes was the dominant phylum, followed by Proteobacteria, Firmicutes, and Bacteroidetes. Navarrete et al., 2013 [59] found that some bacterial groups, such as Shewanella, Lactococcus, Aeromonas, and Pseudomonas were specific to certain diets; however, there was no correlation between SBM-induced inflammation (50% inclusion) and different bacterial groups. The authors also found that the inclusion of lactic acid bacteria may modulate intestinal inflammation. Finally, the inclusion of 5–10% of soybean protein concentrate in the diet did not show better results; Green et al. [75] confirmed the hypothesis that soybean protein concentrate can cause intestinal disorders as a consequence of the intestinal microbiota alteration, even if the authors did not observe any sign of inflammation. The authors also described that soybean protein concentrate could have an effect on raising the bacterial diversity of the intestinal tract, leading to the presence of unusual bacteria, like Escherichia and Propionibacterium, that are not usually associated with marine fish. The impact of yeast inclusion on the modulation of the intestinal microbiota of fish fed SBM was studied by Agboola et al. [77], and it was concluded that the addition of inactivated yeasts did not lead to an alteration in the modulation caused by the SBM diet.

### 3.2. Peas and Pea Protein Concentrate (PPC)

Despite a lower content of protein when compared to other plant protein sources, field peas seem to be a good candidate as a protein source for fish [45,46,47]. Peas also contain several anti-nutritional factors, but their level is low compared with other legumes [35]. Supplementation with peas has been tested in Atlantic salmon and its effect was assessed in terms of intestine integrity.

Intestine health—no effects: No histological changes were observed in the DI of Atlantic salmon fed extruded whole field peas at an inclusion level of 20% [45]. A study showed that PPC could replace 20% of the FM protein in the feed for Atlantic salmon without adverse effects on intestine health; no changes in the size of the intestine and no significant differences in the histology of the DI among dietary groups were detected [61]. These results were recently confirmed by [62], where in fish fed a PPC diet at an inclusion level of 4%, there were no signs of DI enteritis.

Intestine health—negative effects: Penn et al. [43] tested several diets with different inclusion levels of plant proteins. In fish-fed diets containing 35% of PPC, there were evident histological changes in the DI: shorter simple mucosal folds, wider lamina propria with concomitant leukocyte infiltration, reduced to absent vacuolization, and apical displacement of nuclei. It was also evident that there was a reduction in the brush border enzyme activities.

Microbiota: Only one work analyzing the relationship between PPC and microbiota was found in the literature. Hartviksen [66] observed an increase in the population level of autochthonous bacteria associated with the ID mucosa, although the reason for this was not clear. In all diets, the dominant phyla were Corynebacteriaceae in the allochthonous population and Lactobacillaceae in the autochthonous bacterial population. The same authors found no serious signs of enteritis.

### 3.3. Faba Bean and Faba Bean Protein Concentrate (BPC)

Whole crushed faba beans have a lower protein content (~25%) when compared with soybean (~35%) [94]. As for peas, further faba bean processing may lead to ingredients with a higher protein concentration, more interesting for FM replacement: crushed faba beans (28% protein) and air-classified, de-hulled, crushed faba bean protein concentrate (BPC, 61% protein).

Intestine health—no effects: No histological changes were observed in the DI of Atlantic salmon fed either whole or dehulled faba beans at an inclusion level of 20% [45].

Intestine health—negative effects: The same author tested the effect of two levels (17 and 35%) of a wet-processed faba bean isolate with a high protein content (~80% crude protein), and low levels of anti-nutritional factors [63]. The DI of fish fed the diet with the highest content of faba bean showed a clear deterioration of the absorptive vacuoles with an increasing level of supplementation of the wet processed faba. The lamina propria appeared significantly wider, which was probably due to the increased infiltration of eosinophilic granulocytes. These signs of progressing enteritis were similar to those observed in SBM-induced enteritis. The authors concluded that protein derived from faba beans may replace FM at a certain level in feeds for Atlantic salmon.

Intestine health—positive effects: Inclusion levels ranging from 50 to 200 g/kg of feed, which partially replaced either SPC or FM, showed the greatest potential in terms of fish growth and no negative effects in terms of intestine inflammation. This was evaluated through a histological scoring of the following parameters: sub-epithelial mucosa, mucosal folds, lamina propria, eosinophilic granulocytes, goblet cells, and supranuclear vacuoles. High inclusion levels of BPC (450 g/kg) caused mild intestinal inflammation, although this was not as severe as the inflammation seen in fish fed higher SBM diets. The authors concluded that BPC at these inclusion levels represents a valuable alternative for FM in Atlantic salmon.

Microbiota: As for the effects of the use of faba beans in diets on intestinal microbiota, no information was found in the literature.

### 3.4. Other Plant Protein Sources (PPS)

Other plant ingredients were evaluated as protein sources: wheat, maize meal, sunflower meal, rapeseed meal, and wheat gluten.

Intestine health—no effects: The addition of a seaweed meal derived from brown seaweeds (*Laminaria* sp., kelp) was tested in Atlantic salmon at inclusion levels from 3 to 100% [95]. Fish fed the 3 or 10% inclusion levels diets showed higher whole intestine and intestinal weights and lengths, suggesting an increased holding time and a larger surface area for digestion and nutrient absorption. Two commercially available algae-derived products (dry algae meal), Verdemin (derived from *Ulva ohnoi*) and Rosamin (derived from diatom *Entomoneis* spp.), were tested in the diet of Atlantic Salmon [96]. No positive or negative effects were found on fish growth, but the authors did not investigate the effects of this algae inclusion on intestine morphology and health.

Intestine health—negative effects: In Atlantic salmon fed 25% of a high plant protein replacement diet, there was inflammatory infiltration in the submucosa and lamina propria of the DI, but at a low level [65]. Wheat gluten was investigated in two inclusion levels in the diet of Atlantic salmon, 15 or 30% [97]. The authors found shifts in the expression of a large number of genes responsible for immunity and tissue structure and the integrity of the intestine transcriptome in fish fed wheat gluten, indicating the nutritional stress of fish. A high wheat gluten inclusion in the diet produced a negative impact on intestinal health, and symptoms were found to be similar to those of gluten sensitivity in humans.

Microbiota: No information was found in the available literature on the impact of these plant protein sources on the intestinal microbiota.

### 3.5. Animal Protein: Poultry by-Product Meal (PBM)

In this review, until now, only plant protein sources have been addressed as replacement ingredients for FM, but there are animal protein sources that may constitute valid alternatives as well. A good example is the poultry by-product meal (PBM), which is a result of the poultry processing industry. It presents a high protein content with a favorable profile of essential amino acids for fish production [98]. These characteristics, together with its low price and wide availability, make PBM an ideal candidate for replacing FM in aquaculture feeds. However, there are not many studies regarding the impact of the replacement of FM with PBM in the intestines of Atlantic salmon.

Intestine health—negative effects: PBM and feather meal were given to Atlantic salmon at an inclusion level of 20% [62]. None of the fish fed these diets showed severe signs of inflammation. It has to be pointed out that PBM resulted in a significantly decreased submucosa width, and feather meal resulted in a decrease in goblet cells.

Intestine health—positive effects: A diet containing 58% of PBM was given to fish in a 40-day trial [67]. There were no changes in the DI histology, but there was a decrease in the chyme water content and an increase in the expression of the tight junction protein occluding. This is a contradictory result and the authors hypothesize that the decrease in the chyme water content was probably related to the low lipid digestibility.

Microbiota: Hartviksen et al. [62] observed that Atlantic salmon fed with PBM and feather meal influenced the microbiota composition of the intestine; it seems that PBM modulates the allochthonous bacteria composition, by increasing its population in the DI. In addition, in this diet, the main represented phyla were *Corynebacteriaceaein* for the allochthonous and *Lactobacillaceaein* for the autochthonous bacteria population.

### 3.6. Animal Protein: Insect Meal (IM)

#### 3.6.1. Black Soldier Fly (BSF-Hermetia Illucens)

There is an animal protein source for which interest as a feed ingredient for terrestrial and aquatic animals continues to grow every year: insect meal (IM). Among these, the black soldier fly (BSF-*Hermetia illucens*) larvae are considered an important potential species to be used for animal feed [99,100]. This species has been used as a protein source in animal feed since the 1970s, mainly because of its capacity to convert food waste, such as vegetables, fruit, factory waste, and animal tissues, into high-quality protein. BSF larvae contain about 40% protein and present a balanced profile of essential amino acids [101].

Intestine health—no effects: Intestine health seemed to be maintained in fish fed a test diet where all the FM was replaced with BSF larvae meal [71]. Fish fed insect meal presented a higher weight of the DI and less severe enterocytes steatosis (abnormal retention of fat in the cells). There were, however, inflammatory morphological changes in all the examined intestinal segments, which are similar to those to those caused by SBM, but, overall, the authors concluded that intestinal health was not compromised by the IM.

Recently, a research group assessed the partial replacement of FM with BSF larvae in Atlantic salmon fry [69] and in pre-smolts [68]. According to the authors, it is safe to replace FM with BSF up to 10% in fry, as no negative effects on growth, feed utilization, and intestine structure were observed; the mucosa folds and thickness, as well as the overall histology, were not influenced by the BSF larvae meal inclusion. According to the authors, for pre-smolts, it is possible to increase the replacement level up to 15% without compromising growth performance, feed utilization, somatic indices, and the histomorphology of the intestine. Indeed, villus height and area, and the muscular thickness of the DI appeared unaltered when using a BSF larvae meal up to 15% inclusion level. The villus width was higher in fish fed the insect meal diet, but the authors claim that it is not necessarily a negative result, as it may imply an increase in the number and measure of the enterocytes, which provide a greater absorption surface area.

Intestine health—positive effects: Belghit et al. [102] studied the effect of the increasing supplementation of IM, produced from BSF larvae, in the diet of Atlantic salmon. The partial or complete replacement of the FM protein with insect meal in the diet did not show negative effects on the feed intake or on the growth performance of fish. Unfortunately, in this study, the histomorphology of the intestine was not evaluated, but the fact that the inclusion of insect meal did not affect the digestibility of proteins, lipids, amino acids, and fatty acids indicates that most probably the intestine architecture was not significantly altered. This is confirmed by other studies where several levels of insect meal produced from BSF larvae were tested on Atlantic salmon. For instance, Weththasinghe et al. [70] showed that replacing conventional protein sources with low to moderate levels of insect meal (6.25% and 12.5%) leads to an evident reduction in enterocyte steatosis in the pyloric caeca. Even with high replacement levels, such as 25%, an improvement in the DI structure was observed; in all of the experimental groups, an increase in the width and infiltration of the submucosa and lamina propria by inflammatory cells was observed. There were no signs of enteritis in the DI in fish fed the IM diet. Likewise, Li et al. [103] confirmed that the pyloric caeca mucosa clearly showed less hyper-vacuolization (indicative of lipid accumulation in enterocytes) in the IM-fed fish. As for the DI, the authors assessed the shortening and fusion of mucosal folds, cellular infiltration within the lamina propria and submucosa, enterocyte vacuolization, and nucleus position disparity. None of these parameters were affected by the inclusion of IM in the diet. Lock et al. [17] noticed that there were no differences in the DI structure between the several inclusion levels of BSF larvae (from 25 to 100%); the DI epithelium showed a normal morphology with no loss of villi architecture, vacuole formation, or necrosis in the tips of the villi.

Microbiota: Another indication of positive use of the BSF was found in a study by Li et al. [78], where Atlantic salmon were fed a commercial reference diet and an IM-based test diet to compare the intestinal microbiota between the two diets. The authors concluded that fish fed the IM diet showed deep changes in the intestinal microbiota, confirming the results of a previous study of the same authors, where an IM diet altered the DI microbiota in terms of higher microbial richness and diversity. It was found that bacterial communities such as *Actinomyces*, *Bacillus*, *Brevibacterium*, *Corynebacterium 1*, and *Enterococcus* were increased in the IM diet [104] The authors state, though, that further studies would be necessary to evaluate if and how much these alterations can be accountable to differences in microbiota feed and dietary nutrients. This could be useful also to understand the impact of these changes on fish physiology and health.

#### 3.6.2. Yellow Mealworm (Tenebrio Molitor)

Another candidate as IM for aquaculture feeds is the yellow mealworm (*Tenebrio molitor*). Like the BSF larvae, the *T. molitor* also has a substantial content of proteins and lipids, and a good amino acid and essential fatty acid profile. Shafique et al. [105] made an interesting review on the use of this insect species in aquafeed; unfortunately, there is no information on Atlantic salmon, but the results obtained up to now in other salmonids seem promising in terms of growth, biometric indices, and body composition. As the authors mention, further studies are necessary in order to conduct a thorough evaluation of the impact of the use of this ingredient in terms of intestine health in fish. The impact on the intestine microbiota from the addition of the yellow mealworm in the diets of Atlantic salmon has not been studied to date.

### 3.7. Single-Cell Protein (SCP)

Single-cell protein (SCP) also appears to be an excellent alternative to FM and to plant ingredients due to its adequate nutritional profile and the fact that it can be cultivated in any location and climate. Moreover, it presents the advantage of not stressing the existing agricultural systems. SCP products can be produced from several microbial sources, such as microalgae, yeast and other fungi, and bacteria [106].

#### 3.7.1. Microalgae

Microalgae are rich in high-quality proteins, essential amino acids, polyunsaturated fatty acids, polysaccharides, vitamins, minerals, and pigments [107].

Intestine health—no effects: The effect of the replacement of FM with the microalga *Nannochloropsis oceania* was assessed in terms of growth performance, feed utilization, and intestine health [108]. The histomorphology of the DI was not altered by the supplementation with this microalga in the feed, and there were no signs of inflammation of the intestine, even if the expression of the pro-inflammatory gene il17d was higher in fish fed 10% alga. The results of this study, altogether, indicate that the inclusion of this microalgae at low levels (c.a. 10%) does not produce negative effects on weight gain, specific growth rate, and health parameters, making it a good candidate for FM partial replacement. The same author examined the effect of pre-extruding two microalgae, *Nannochloropsis oceanica*, and *Tetraselmis* sp., on the growth, the fatty acid content in the flesh, and the health of Atlantic salmon [72]. First of all, neither *Nannochloropsis* nor *Tetraselmis*, at high inclusion levels (30%) show signs of intestine inflammation other than enterocyte vacuolization. For both algae, the expression of *muc2*, a gene that encodes the mucine protein which covers the whole intestine, in the DI was not significantly altered. Microalgae present rigid cell walls that may lower the digestibility and nutrient bioavailability in carnivorous fish, but the extrusion process may contribute to its breakdown and therefore to better nutrient utilization. It has been hypothesized that certain food additives may contribute to a better nutrient utilization of microalgae by fish. Gong et al. [109] tested the influence of two commercial food additives in food containing 10% of *N. oceanica*. The addition of the feed additives produced no significant effects on growth, feed utilization, or the histomorphology of the DI. Even if there was an increase in the cell proliferation of the mucosa folds in fish fed the microalga alone, as well as the microalga-commercial additive combination, the authors concluded that the tested feed additives did not show a distinct advantage for Atlantic salmon.

Intestine health—positive effects: Kiron et al. [73] investigated the inclusion of a defatted marine alga, the *Desmodesmus* sp., in Atlantic salmon diets. The expression of immune and inflammatory marker genes and the morphological observations did not reveal any abnormalities in the intestinal health of the fish fed microalga. Histological analyses revealed a normal lamina propria and goblet cells. The authors concluded that it is possible to include defatted *Desmodesmus* sp. at 20% in the feeds of Atlantic salmon. Grammes et al. [110] investigated the possibility that the addition of certain microbial ingredients such as yeasts or microalgae may relieve SBM-induced enteritis in Atlantic salmon. The results showed that the supplementation of a 20% SBM feed with the yeast *Candida utilis* or the microalgae *Chlorella vulgaris* resulted in a healthy fish intestine, indicating it was highly effective to counteract enteritis.

#### 3.7.2. Yeasts

Yeasts are becoming popular as ingredients in fish feeds because of their protein content but also because they potentially support better growth and good fish health, as reviewed by Agboola et al. [111].

Intestine health—no effects: The inclusion of 25% *Candida utilis* yeast in the feed during the delicate stage of smoltification did not produce any alterations in the morphology of the DI, in terms of the length of the simple folds, mucosa thickness, or intestinal epithelial cells [23]. The replacement of 40% of the crude protein from FM with the yeasts *Candida utilis*, *Kluyveromyces marxianus*, or *Saccharomyces cerevisiae* was evaluated [112]. Fish fed the *S. cerevisiae* and *K. marxianus* diets presented a heavier DI than fish fed the FM diet, but no other assessments were performed on the intestine. Overall, the authors concluded that *C. utilis* and *K. marxianus* can be used as suitable protein sources for Atlantic salmon, while *S. cerevisiae* seems to be less promising.

Reveco-Urzua et al. [53] aimed to evaluate the intestinal tissue responses of fish fed inactive dry *Candida utilis* yeast biomass, SBM, or a combination of an SBM-based diet with several inclusion levels of *Candida utilis.* The authors found that the inclusion of *C. utilis* in an FM-based diet did not produce any changes in terms of DI morphology, immune cell population, or gene expression. When combined with SBM, low inclusion levels of *C. utilis* modulated immune cell populations in the DI and reduced the severity of SBM-induced enteritis. However, this effect on enteritis was not confirmed by a study on parr, where the severity of the reduced height of the intestine folds and the decreased presence of supranuclear vacuoles was not counteracted by the addition of *C. utilis* in the feed [74].

Microbiota: It has been observed that an inclusion level of up to 20% of torula yeast in Atlantic salmon feed produced no alterations in growth performance, and showed benefits in the intestine microbial community [79].

However, at inclusion levels higher than 20%, not only were there no growth benefits, but there were adverse changes in the intestine microbiome, for example, an increase in the bacterial community, such as *Weissella* and *Leuconostoc*, and also a decrease in lactic acid bacteria (*Lactococcus*).

## 4. Conclusions

The development of more sustainable aquaculture from the economic and environmental point of view should be focused on the development of diets with new, sustainable sources of proteins to fully or partially replace fishmeal. The future elected protein sources must meet the nutritional requirements of carnivorous fish species such as the Atlantic salmon without compromising the growth and intestine health of fish, which are usually endorsed by the anti-nutritional factors present in many plant ingredients. It appears quite evident from this review that even at low inclusion levels, soybean meal causes negative effects on the distal intestine of Atlantic salmon. Nevertheless, soybean meal remains an attractive alternative to fishmeal, taking into account its protein content, market availability, and cost, although other protein sources should be seriously taken into account when formulating feeds. Ingredients with an adequate content of protein, which cause mild or no effects on the distal intestine even at high inclusion levels, such as microalgae, insects, and single-cell proteins, could be also interesting choices for feed formulations.

The negative effects on morphology caused by alternative protein sources seem to be greater than the positive ones. However, concerning carnivorous fish such as the Atlantic salmon, if an alternative protein source produces no negative effects on fish health, then this is an overall positive effect from a sustainability point of view. This review shows that this is a “hot topic” nowadays. There is a lot of research on new sustainable protein sources, but this research is not always easy to confront as there are a great variety of inclusion levels, trial lengths, and stages of fish development. In the current scenario, more research still needs to be performed in order to reach a balanced alternative to fishmeal, with the right inclusion level used in the right fish stage of development. Indeed, regarding microbiota, it is now starting to be studied, and from this review it can be seen that there are not many works yet and therefore it is not yet possible to draw clear conclusions.

Finally, using alternative protein sources could have a positive impact, as well, on the preservation of the stocks of marine species used for the production of fishmeal. It may not be ethically correct to harvest fish that is used to produce aquaculture feed, to produce fish that will be used as food for humans [39,113]. Thus, it is of major importance to increase efforts to replace fishmeal in feeds for fish in general, and for Atlantic salmon in particular, and to pursue strategies that minimize the impacts of fishmeal reduction in the diet and on fish fillet quality. The alternative protein sources in aquafeeds must guarantee fish health but also a final product that is suitable from a nutritional point of view, and at the same time well accepted by the consumers.

## References

[1] Løkka G., Austbø L., Falk K., Bjerkås I., Koppang E.O. (2013). Intestinal Morphology of the Wild Atlantic Salmon (*Salmo salar*). J. Morphol..

[2] Kapoor B.G., Smit H., Verighina I.A., Russell F.S., Yonge M. (1976). The Alimentary Canal and Digestion in Teleosts. Advances in Marine Biology.

[3] Bjørgen H., Li Y., Kortner T.M., Krogdahl Å., Koppang E.O. (2020). Anatomy, Immunology, Digestive Physiology and Microbiota of the Salmonid Intestine: Knowns and Unknowns under the Impact of an Expanding Industrialized Production. Fish Shellfish Immunol..

[4] Harder W. (1975). The Digestive Tract. Anatomy of Fishes. Part 1: Text.

[5] Rust M.B., Halver J.E., Hardy R.W. (2003). 7—Nutritional Physiology. Fish Nutrition.

[6] Denstadli V., Vegusdal A., Krogdahl Å., Bakke-McKellep A.M., Berge G.M., Holm H., Hillestad M., Ruyter B. (2004). Lipid Absorption in Different Segments of the Gastrointestinal Tract of Atlantic Salmon (*Salmo salar* L.). Aquaculture.

[7] Burnstock G. (1959). The Morphology of the Gut of the Brown Trout (*Salmo trutta*). J. Cell Sci..

[8] Sundell K., Jutfelt F., Ágústsson T., Olsen R.-E., Sandblom E., Hansen T., Björnsson B.T. (2003). Intestinal Transport Mechanisms and Plasma Cortisol Levels during Normal and Out-of-Season Parr–Smolt Transformation of Atlantic Salmon. Salmo salar. Aquac..

[9] Sire M.F., Vernier J.-M. (1992). Intestinal Absorption of Protein in Teleost Fish. Comp. Biochem. Physiol. A Physiol..

[10] Kent G., Carr R., Kent G.C., Carr R.K. (2001). Digestive System. Comparative Anatomy of the Vertebrates.

[11] Jutfelt F. (2011). Barrier Function of the Gut. Encyclopedia of Fish Physiology from Genome to Environment.

[12] Takei Y., Loretz C.A., Grosell M., Farrell A.P., Brauner C.J. (2010). 7—The Gastrointestinal Tract as an Endocrine/Neuroendocrine/Paracrine Organ: Organization, Chemical Messengers and Physiological Targets. Fish Physiology.

[13] Wilson J.M., Castro L.F.C., Grosell M., Farrell A.P., Brauner C.J. (2010). 1—Morphological Diversity of the Gastrointestinal Tract in Fishes. Fish Physiology.

[14] Grosell M. (2011). Intestinal Anion Exchange in Marine Teleosts Is Involved in Osmoregulation and Contributes to the Oceanic Inorganic Carbon Cycle. Acta Physiol..

[15] Pascoli F., Negrato E., Di Giancamillo A., Bertotto D., Domeneghini C., Simontacchi C., Mutinelli F., Radaelli G. (2011). Evaluation of Oxidative Stress Biomarkers in Zosterisessor Ophiocephalus from the Venice Lagoon, Italy. Aquat. Toxicol..

[16] Poussier P., Edouard P., Lee C., Binnie M., Julius M. (1992). Thymus-Independent Development and Negative Selection of T Cells Expressing T Cell Receptor Alpha/Beta in the Intestinal Epithelium: Evidence for Distinct Circulation Patterns of Gut- and Thymus-Derived T Lymphocytes. J. Exp. Med..

[17] Lock E.R., Arsiwalla T., Waagbø R. (2016). Insect Larvae Meal as an Alternative Source of Nutrients in the Diet of Atlantic Salmon (*Salmo salar*) Postsmolt. Aquac. Nutr..

[18] Bjørgen H., Hellberg H., Løken O.M., Gunnes G., Koppang E.O., Dale O.B. (2019). Tumor Microenvironment and Stroma in Intestinal Adenocarcinomas and Associated Metastases in Atlantic Salmon Broodfish (*Salmo salar*). Vet. Immunol. Immunopathol..

[19] Tafalla C., Leal E., Yamaguchi T., Fischer U. (2016). T Cell Immunity in the Teleost Digestive Tract. Dev. Comp. Immunol..

[20] Ng A.N.Y., de Jong-Curtain T.A., Mawdsley D.J., White S.J., Shin J., Appel B., Dong P.D.S., Stainier D.Y.R., Heath J.K. (2005). Formation of the Digestive System in Zebrafish: III. Intestinal Epithelium Morphogenesis. Dev. Biol..

[21] Wallace K.N., Akhter S., Smith E.M., Lorent K., Pack M. (2005). Intestinal Growth and Differentiation in Zebrafish. Mech. Dev..

[22] Whittamore J.M. (2012). Osmoregulation and Epithelial Water Transport: Lessons from the Intestine of Marine Teleost Fish. J. Comp. Physiol. B.

[23] Sahlmann C., Djordjevic B., Lagos L., Mydland L.T., Morales-Lange B., Øvrum Hansen J., Ånestad R., Mercado L., Bjelanovic M., Press C.M. (2019). Yeast as a Protein Source during Smoltification of Atlantic Salmon (*Salmo salar* L.), Enhances Performance and Modulates Health. Aquaculture.

[24] Dhanasiri A.K.S., Brunvold L., Brinchmann M.F., Korsnes K., Bergh Ø., Kiron V. (2011). Changes in the Intestinal Microbiota of Wild Atlantic Cod *Gadus Morhua* L. Upon Captive Rearing. Microb. Ecol..

[25] Ringø E., Zhou Z., Vecino J.L.G., Wadsworth S., Romero J., Krogdahl Å., Olsen R.e., Dimitroglou A., Foey A., Davies S. (2016). Effect of Dietary Components on the Gut Microbiota of Aquatic Animals. A Never-Ending Story?. Aquac. Nutr..

[26] Ringø E., Olsen R.E., Mayhew T.M., Myklebust R. (2003). Electron Microscopy of the Intestinal Microflora of Fish. Aquaculture.

[27] Wang J., Jaramillo-Torres A., Li Y., Kortner T.M., Gajardo K., Brevik Ø.J., Jakobsen J.V., Krogdahl Å. (2021). Microbiota in Intestinal Digesta of Atlantic Salmon (*Salmo salar*), Observed from Late Freshwater Stage until One Year in Seawater, and Effects of Functional Ingredients: A Case Study from a Commercial Sized Research Site in the Arctic Region. Anim. Microbiome.

[28] Falcinelli S., Picchietti S., Rodiles A., Cossignani L., Merrifield D.L., Taddei A.R., Maradonna F., Olivotto I., Gioacchini G., Carnevali O. (2015). *Lactobacillus Rhamnosus* Lowers Zebrafish Lipid Content by Changing Gut Microbiota and Host Transcription of Genes Involved in Lipid Metabolism. Sci. Rep..

[29] Semova I., Carten J.D., Stombaugh J., Mackey L.C., Knight R., Farber S.A., Rawls J.F. (2012). Microbiota Regulate Intestinal Absorption and Metabolism of Fatty Acids in the Zebrafish. Cell Host Microbe.

[30] Ray A.K., Ghosh K., Ringø E. (2012). Enzyme-Producing Bacteria Isolated from Fish Gut: A Review. Aquac. Nutr..

[31] Austin B. (2006). The Bacterial Microflora of Fish, Revised. Sci. World J..

[32] López Nadal A., Ikeda-Ohtsubo W., Sipkema D., Peggs D., McGurk C., Forlenza M., Wiegertjes G.F., Brugman S. (2020). Feed, Microbiota, and Gut Immunity: Using the Zebrafish Model to Understand Fish Health. Front. Immunol..

[33] FAO: Nutritional Requirements. https://www.fao.org/fishery/affris/species-profiles/atlantic-salmon/nutritional-requirements/en/.

[34] Lall S.P., Anderson S. (2005). Amino Acid Nutrition of Salmonids: Dietary Requirements and Bioavailablity. Cah. Options Mediterr..

[35] Francis G., Makkar H.P.S., Becker K. (2001). Antinutritional Factors Present in Plant-Derived Alternate Fish Feed Ingredients and Their Effects in Fish. Aquaculture.

[36] Gatlin D.M., Barrows F.T., Brown P., Dabrowski K., Gaylord T.G., Hardy R.W., Herman E., Hu G., Krogdahl Å., Nelson R. (2007). Expanding the Utilization of Sustainable Plant Products in Aquafeeds: A Review. Aquac. Res..

[37] FAO (Food and Agriculture Organization of the United Nations) Fisheries and Aquaculture Department—Species Fact Sheets. www.fao.org.

[38] Crespi V. (2020). The State of World Fisheries and Aquaculture.

[39] Tacon A.G.J., Metian M. (2008). Global Overview on the Use of Fish Meal and Fish Oil in Industrially Compounded Aquafeeds: Trends and Future Prospects. Aquaculture.

[40] Gillund F., Myhr A.I. (2010). Perspectives on Salmon Feed: A Deliberative Assessment of Several Alternative Feed Resources. J. Agric. Environ. Ethics.

[41] Bakke-McKellep A.M., Koppang E.O., Gunnes G., Sanden M., Hemre G.-I., Landsverk T., Krogdahl A. (2007). Histological, Digestive, Metabolic, Hormonal and Some Immune Factor Responses in Atlantic Salmon, *Salmo salar* L., Fed Genetically Modified Soybeans. J. Fish Dis..

[42] Dale O.B., Tørud B., Kvellestad A., Koppang H.S., Koppang E.O. (2009). From Chronic Feed-Induced Intestinal Inflammation to Adenocarcinoma with Metastases in Salmonid Fish. Cancer Res..

[43] Penn M.H., Bendiksen E.Å., Campbell P., Krogdahl Å. (2011). High Level of Dietary Pea Protein Concentrate Induces Enteropathy in Atlantic Salmon (*Salmo salar* L.). Aquaculture.

[44] Urán P.A., Schrama J.W., Rombout J.H.W.M., Obach A., Jensen L., Koppe W., Verreth J.A.J. (2008). Soybean Meal-Induced Enteritis in Atlantic Salmon (*Salmo salar* L.) at Different Temperatures. Aquac. Nutr..

[45] Aslaksen M.A., Kraugerud O.F., Penn M., Svihus B., Denstadli V., Jørgensen H.Y., Hillestad M., Krogdahl Å., Storebakken T. (2007). Screening of Nutrient Digestibilities and Intestinal Pathologies in Atlantic Salmon, *Salmo salar*, Fed Diets with Legumes, Oilseeds, or Cereals. Aquaculture.

[46] Thiessen D.L., Campbell G.L., Adelizi P.D. (2003). Digestibility and Growth Performance of Juvenile Rainbow Trout (*Oncorhynchus Mykiss*) Fed with Pea and Canola Products. Aquac. Nutr..

[47] Gouveia A., Davies S.J. (2000). Inclusion of an Extruded Dehulled Pea Seed Meal in Diets for Juvenile European Sea Bass (*Dicentrarchus Labrax*). Aquaculture.

[48] Shubin A.V., Demidyuk I.V., Komissarov A.A., Rafieva L.M., Kostrov S.V. (2016). Cytoplasmic Vacuolization in Cell Death and Survival. Oncotarget.

[49] Gajardo K., Jaramillo-Torres A., Kortner T.M., Merrifield D.L., Tinsley J., Bakke A.M., Krogdahl Å. (2016). Alternative Protein Sources in the Diet Modulate Microbiota and Functionality in the Distal Intestine of Atlantic Salmon (*Salmo salar*). Appl. Environ. Microbiol..

[50] Krogdahl Å., Bakke-McKellep A.M., Baeverfjord G. (2003). Effects of Graded Levels of Standard Soybean Meal on Intestinal Structure, Mucosal Enzyme Activities, and Pancreatic Response in Atlantic Salmon (*Salmo salar* L.). Aquac. Nutr..

[51] Urán P.A., Schrama J.W., Rombout J.H.W.M., Taverne-Thiele J.J., Obach A., Koppe W., Verreth J. (2009). a J. Time-Related Changes of the Intestinal Morphology of Atlantic Salmon, *Salmo salar* L., at Two Different Soybean Meal Inclusion Levels. J. Fish Dis..

[52] Booman M., Forster I., Vederas J.C., Groman D.B., Jones S.R.M. (2018). Soybean Meal-Induced Enteritis in Atlantic Salmon (*Salmo salar*) and Chinook Salmon (*Oncorhynchus tshawytscha*) but Not in Pink Salmon (*O. Gorbuscha*). Aquaculture.

[53] Reveco-Urzua F.E., Hofossæter M., Kovi M.R., Mydland L.T., Ånestad R., Sørby R., Press C.M., Lagos L., Øverland M. (2019). Candida Utilis Yeast as a Functional Protein Source for Atlantic Salmon (*Salmo salar* L.): Local Intestinal Tissue and Plasma Proteome Responses. PLoS ONE.

[54] Nimalan N., Sørensen S.L., Fečkaninová A., Koščová J., Mudroňová D., Gancarčíková S., Vatsos I.N., Bisa S., Kiron V., Sørensen M. (2023). Supplementation of Lactic Acid Bacteria Has Positive Effects on the Mucosal Health of Atlantic Salmon (*Salmo salar*) Fed Soybean Meal. Aquac. Rep..

[55] Nordrum S., Bakke-McKellep A.M., Krogdahl A., Buddington R.K. (2000). Effects of Soybean Meal and Salinity on Intestinal Transport of Nutrients in Atlantic Salmon (*Salmo salar* L.) and Rainbow Trout (*Oncorhynchus mykiss*). Comp. Biochem. Physiol. B Biochem. Mol. Biol..

[56] Refstie S., Baeverfjord G., Seim R.R., Elvebø O. (2010). Effects of Dietary Yeast Cell Wall β-Glucans and MOS on Performance, Gut Health, and Salmon Lice Resistance in Atlantic Salmon (*Salmo salar*) Fed Sunflower and Soybean Meal. Aquaculture.

[57] Baeverfjord G., Krogdahl A. (1996). Development and Regression of Soybean Meal Induced Enteritis in Atlantic Salmon, *Salmo salar* L., Distal Intestine: A Comparison with the Intestines of Fasted Fish. J. Fish Dis..

[58] Bakke-McKellep A.M., Press C.M., Baeverfjord G., Krogdahl Å., Landsverk T. (2000). Changes in Immune and Enzyme Histochemical Phenotypes of Cells in the Intestinal Mucosa of Atlantic Salmon, *Salmo salar* L., with Soybean Meal-Induced Enteritis. J. Fish Dis..

[59] Navarrete P., Fuentes P., la Fuente L.D., Barros L., Magne F., Opazo R., Ibacache C., Espejo R., Romero J. (2013). Short-Term Effects of Dietary Soybean Meal and Lactic Acid Bacteria on the Intestinal Morphology and Microbiota of Atlantic Salmon (*Salmo salar*). Aquac. Nutr..

[60] Nordvi M.F., Løvmo S.D., Whatmore P., Sundh H., Sigholt T., Olsen R.E. (2023). Low Intestinal Inflammation Model (HP48) in Atlantic Salmon (*Salmo salar*) and Inflammatory Mitigation by Bactocell. Aquaculture.

[61] Øverland M., Sørensen M., Storebakken T., Penn M., Krogdahl Å., Skrede A. (2009). Pea Protein Concentrate Substituting Fish Meal or Soybean Meal in Diets for Atlantic Salmon (*Salmo salar*)—Effect on Growth Performance, Nutrient Digestibility, Carcass Composition, Gut Health, and Physical Feed Quality. Aquaculture.

[62] Hartviksen M., Vecino J.L.G., Ringø E., Bakke A.-M., Wadsworth S., Krogdahl Å., Ruohonen K., Kettunen A. (2014). Alternative Dietary Protein Sources for Atlantic Salmon (*Salmo salar* L.) Effect on Intestinal Microbiota, Intestinal and Liver Histology and Growth. Aquac. Nutr..

[63] De Santis C., Martin S.A.M., Dehler C.E., Iannetta P.P.M., Leeming D., Tocher D.R. (2016). Influence of Dietary Inclusion of a Wet Processed Faba Bean Protein Isolate on Post-Smolt Atlantic Salmon (*Salmo salar*). Aquaculture.

[64] De Santis C., Ruohonen K., Tocher D.R., Martin S.A.M., Król E., Secombes C.J., Bell J.G., El-Mowafi A., Crampton V.O. (2015). Atlantic Salmon (*Salmo salar*) Parr as a Model to Predict the Optimum Inclusion of Air Classified Faba Bean Protein Concentrate in Feeds for Seawater Salmon. Aquaculture.

[65] Tacchi L., Secombes C.J., Bickerdike R., Adler M.A., Venegas C., Takle H., Martin S.A. (2012). Transcriptomic and Physiological Responses to Fishmeal Substitution with Plant Proteins in Formulated Feed in Farmed Atlantic Salmon (*Salmo salar*). BMC Genom..

[66] Hartviksen M., Bakke A.M., Vecino J.G., Ringø E., Krogdahl Å. (2014). Evaluation of the Effect of Commercially Available Plant and Animal Protein Sources in Diets for Atlantic Salmon (*Salmo salar* L.): Digestive and Metabolic Investigations. Fish Physiol. Biochem..

[67] Hu H., Kortner T.M., Gajardo K., Chikwati E., Tinsley J., Krogdahl Å. (2016). Intestinal Fluid Permeability in Atlantic Salmon (*Salmo salar* L.) Is Affected by Dietary Protein Source. PLoS ONE.

[68] Mikołajczak Z., Mazurkiewicz J., Rawski M., Kierończyk B., Józefiak A., Świątkiewicz S., Józefiak D. (2022). Black Soldier Fly Full-Fat Meal in Atlantic Salmon Nutrition—Part B: Effects on Growth Performance, Feed Utilization, Selected Nutriphysiological Traits and Production Sustainability in Pre-Smolts. Ann. Anim. Sci..

[69] Mikołajczak Z., Mazurkiewicz J., Rawski M., Kierończyk B., Józefiak A., Świątkiewicz S., Józefiak D. (2022). Black Soldier Fly Full-Fat Meal in Atlantic Salmon Nutrition—Part A: Effects on Growth Performance, Feed Utilization, Selected Nutriphysiological Traits and Production Sustainability in Fries. Ann. Anim. Sci..

[70] Weththasinghe P., Lagos L., Cortés M., Hansen J.Ø., Øverland M. (2021). Dietary Inclusion of Black Soldier Fly (*Hermetia illucens*) Larvae Meal and Paste Improved Gut Health but Had Minor Effects on Skin Mucus Proteome and Immune Response in Atlantic Salmon (*Salmo salar*). Front. Immunol..

[71] Li Y., Kortner T.M., Chikwati E.M., Belghit I., Lock E.-J., Krogdahl Å. (2020). Total Replacement of Fish Meal with Black Soldier Fly (*Hermetia Illucens*) Larvae Meal Does Not Compromise the Gut Health of Atlantic Salmon (*Salmo salar*). Aquaculture.

[72] Sørensen S.L., Ghirmay A., Gong Y., Dahle D., Vasanth G., Sørensen M., Kiron V. (2021). Growth, Chemical Composition, Histology and Antioxidant Genes of Atlantic Salmon (*Salmo salar*) Fed Whole or Pre-Processed *Nannochloropsis Oceanica* and *Tetraselmis* Sp. Fishes.

[73] Kiron V., Sørensen M., Huntley M., Vasanth G.K., Gong Y., Dahle D., Palihawadana A.M. (2016). Defatted Biomass of the Microalga, Desmodesmus Sp., Can Replace Fishmeal in the Feeds for Atlantic Salmon. Front. Mar. Sci..

[74] Hansen J., Hofossæter M., Sahlmann C., Ånestad R., Reveco-Urzua F.E., Press C.M., Mydland L.T., Øverland M. (2019). Effect of Candida Utilis on Growth and Intestinal Health of Atlantic Salmon (*Salmo salar*) Parr. Aquaculture.

[75] Green T.J., Smullen R., Barnes A.C. (2013). Dietary Soybean Protein Concentrate-Induced Intestinal Disorder in Marine Farmed Atlantic Salmon, *Salmo salar* Is Associated with Alterations in Gut Microbiota. Vet. Microbiol..

[76] Reveco F.E., Øverland M., Romarheim O.H., Mydland L.T. (2014). Intestinal Bacterial Community Structure Differs between Healthy and Inflamed Intestines in Atlantic Salmon (*Salmo salar* L.). Aquaculture.

[77] Agboola J.O., Rocha S.D.C., Mensah D.D., Hansen J.Ø., Øyås O., Lapeña D., Mydland L.T., Arntzen M.Ø., Horn S.J., Øverland M. (2023). Effect of Yeast Species and Processing on Intestinal Microbiota of Atlantic Salmon (*Salmo salar*) Fed Soybean Meal-Based Diets in Seawater. Anim. Microbiome.

[78] Li Y., Gajardo K., Jaramillo-Torres A., Kortner T.M., Krogdahl Å. (2022). Consistent Changes in the Intestinal Microbiota of Atlantic Salmon Fed Insect Meal Diets. Anim. Microbiome.

[79] Leeper A., Ekmay R., Knobloch S., Skírnisdóttir S., Varunjikar M., Dubois M., Smárason B.Ö., Árnason J., Koppe W., Benhaïm D. (2022). Torula Yeast in the Diet of Atlantic Salmon *Salmo salar* and the Impact on Growth Performance and Gut Microbiome. Sci. Rep..

[80] Urán P.A., Aydin R., Schrama J.W., Verreth J.A.J., Rombout J.H.W.M. (2008). Soybean Meal-Induced Uptake Block in Atlantic Salmon *Salmo salar* Distal Enterocytes. J. Fish Biol..

[81] Bruce T.J., Neiger R.D., Brown M.L. (2018). Gut Histology, Immunology and the Intestinal Microbiota of Rainbow Trout, *Oncorhynchus Mykiss* (Walbaum), Fed Process Variants of Soybean Meal. Aquac. Res..

[82] Miao S., Zhao C., Zhu J., Hu J., Dong X., Sun L. (2018). Dietary Soybean Meal Affects Intestinal Homoeostasis by Altering the Microbiota, Morphology and Inflammatory Cytokine Gene Expression in Northern Snakehead. Sci. Rep..

[83] Merrifield D.L., Dimitroglou A., Bradley G., Baker R.T.M., Davies S.J. (2009). Soybean Meal Alters Autochthonous Microbial Populations, Microvilli Morphology and Compromises Intestinal Enterocyte Integrity of Rainbow Trout, *Oncorhynchus mykiss* (Walbaum). J. Fish Dis..

[84] Buttle L.G., Burrells A.C., Good J.E., Williams P.D., Southgate P.J., Burrells C. (2001). The Binding of Soybean Agglutinin (SBA) to the Intestinal Epithelium of Atlantic Salmon, *Salmo salar* and Rainbow Trout, Oncorhynchus Mykiss, Fed High Levels of Soybean Meal. Vet. Immunol. Immunopathol..

[85] Gajardo K., Rodiles A., Kortner T.M., Krogdahl Å., Bakke A.M., Merrifield D.L., Sørum H. (2016). A High-Resolution Map of the Gut Microbiota in Atlantic Salmon (*Salmo salar*): A Basis for Comparative Gut Microbial Research. Sci. Rep..

[86] Sanden M., Berntssen M.H.G., Krogdahl Å., Hemre G.-I., Bakke-McKellep A.-M. (2005). An Examination of the Intestinal Tract of Atlantic Salmon, *Salmo salar* L., Parr Fed Different Varieties of Soy and Maize. J. Fish Dis..

[87] Krogdahl, Bakke-Mckellep, RØed, Baeverfjord (2000). Feeding Atlantic Salmon *Salmo salar* L. Soybean Products: Effects on Disease Resistance (Furunculosis), and Lysozyme and IgM Levels in the Intestinal Mucosa. Aquac. Nutr..

[88] Sørensen S.L., Park Y., Gong Y., Vasanth G.K., Dahle D., Korsnes K., Phuong T.H., Kiron V., Øyen S., Pittman K. (2021). Nutrient Digestibility, Growth, Mucosal Barrier Status, and Activity of Leucocytes From Head Kidney of Atlantic Salmon Fed Marine- or Plant-Derived Protein and Lipid Sources. Front. Immunol..

[89] Knudsen D., Urán P., Arnous A., Koppe W., FRØKIÆR† H. (2007). Saponin-Containing Subfractions of Soybean Molasses Induce Enteritis in the Distal Intestine of Atlantic Salmon. J. Agric. Food Chem..

[90] Nikmaram N., Leong S.Y., Koubaa M., Zhu Z., Barba F.J., Greiner R., Oey I., Roohinejad S. (2017). Effect of Extrusion on the Anti-Nutritional Factors of Food Products: An Overview. Food Control..

[91] Catalán N., Villasante A., Wacyk J., Ramírez C., Romero J. (2018). Fermented Soybean Meal Increases Lactic Acid Bacteria in Gut Microbiota of Atlantic Salmon (*Salmo salar*). Probiotics Antimicrob. Proteins.

[92] Krogdahl Å., Kortner T.M., Jaramillo-Torres A., Gamil A.A.A., Chikwati E., Li Y., Schmidt M., Herman E., Hymowitz T., Teimouri S. (2020). Removal of Three Proteinaceous Antinutrients from Soybean Does Not Mitigate Soybean-Induced Enteritis in Atlantic Salmon (*Salmo salar*, L). Aquaculture.

[93] Bakke-McKellep A.M., Penn M.H., Salas P.M., Refstie S., Sperstad S., Landsverk T., Ringø E., Krogdahl Å. (2007). Effects of Dietary Soyabean Meal, Inulin and Oxytetracycline on Intestinal Microbiota and Epithelial Cell Stress, Apoptosis and Proliferation in the Teleost Atlantic Salmon (*Salmo salar* L.). Br. J. Nutr..

[94] El-Shemy H., Abdel-Rahim E., Shaban O., Ragab A., Carnovale E., Fujita K. (2000). Comparison of Nutritional and Antinutritional Factors in Soybean and Fababean Seeds with or without Cortex. Soil Sci. Plant Nutr..

[95] Kamunde C., Sappal R., Melegy T.M. (2019). Brown Seaweed (AquaArom) Supplementation Increases Food Intake and Improves Growth, Antioxidant Status and Resistance to Temperature Stress in Atlantic Salmon, *Salmo salar*. PLoS ONE.

[96] Norambuena F., Hermon K., Skrzypczyk V., Emery J.A., Sharon Y., Beard A., Turchini G.M. (2015). Algae in Fish Feed: Performances and Fatty Acid Metabolism in Juvenile Atlantic Salmon. PLoS ONE.

[97] Johny A., Berge G.M., Bogevik A.S., Krasnov A., Ruyter B., Fæste C.K., Østbye T.-K.K. (2020). Sensitivity to Dietary Wheat Gluten in Atlantic Salmon Indicated by Gene Expression Changes in Liver and Intestine. Genes.

[98] Rawles S.D., Thompson K.R., Brady Y.J., Metts L.S., Aksoy M.Y., Gannam A.L., Twibell R.G., Ostrand S., Webster C.D. (2011). Effects of Replacing Fish Meal with Poultry By-Product Meal and Soybean Meal and Reduced Protein Level on the Performance and Immune Status of Pond-Grown Sunshine Bass (*Morone chrysops* × *M. Saxatilis*). Aquac. Nutr..

[99] Cammack J., Tomberlin J. (2017). The Impact of Diet Protein and Carbohydrate on Select Life-History Traits of The Black Soldier Fly *Hermetia Illucens* (L.) (Diptera: *Stratiomyidae*). Insects.

[100] van Huis A. (2013). Potential of Insects as Food and Feed in Assuring Food Security. Annu. Rev. Entomol..

[101] Wang Y.-S., Shelomi M. (2017). Review of Black Soldier Fly (*Hermetia illucens*) as Animal Feed and Human Food. Foods.

[102] Belghit I., Liland N.S., Gjesdal P., Biancarosa I., Menchetti E., Li Y., Waagbø R., Krogdahl Å., Lock E.-J. (2019). Black Soldier Fly Larvae Meal Can Replace Fish Meal in Diets of Sea-Water Phase Atlantic Salmon (*Salmo salar*). Aquaculture.

[103] Li Y., Kortner T.M., Chikwati E.M., Munang’andu H.M., Lock E.-J., Krogdahl Å. (2019). Gut Health and Vaccination Response in Pre-Smolt Atlantic Salmon (*Salmo salar*) Fed Black Soldier Fly (*Hermetia illucens*) Larvae Meal. Fish Shellfish Immunol..

[104] Li Y., Bruni L., Jaramillo-Torres A., Gajardo K., Kortner T.M., Krogdahl Å. (2021). Differential Response of Digesta- and Mucosa-Associated Intestinal Microbiota to Dietary Insect Meal during the Seawater Phase of Atlantic Salmon. Anim. Microbiome.

[105] Shafique L., Abdel-Latif H.M.R., Hassan F., Alagawany M., Naiel M.A.E., Dawood M.A.O., Yilmaz S., Liu Q. (2021). The Feasibility of Using Yellow Mealworms (*Tenebrio molitor*): Towards a Sustainable Aquafeed Industry. Animals.

[106] Jones S.W., Karpol A., Friedman S., Maru B.T., Tracy B.P. (2020). Recent Advances in Single Cell Protein Use as a Feed Ingredient in Aquaculture. Curr. Opin. Biotechnol..

[107] Becker W. (2003). Microalgae in Human and Animal Nutrition. Handbook of Microalgal Culture.

[108] Sørensen M., Gong Y., Bjarnason F., Vasanth G.K., Dahle D., Huntley M., Kiron V. (2017). Nannochloropsis Oceania-Derived Defatted Meal as an Alternative to Fishmeal in Atlantic Salmon Feeds. PLoS ONE.

[109] Gong Y., Sørensen S.L., Dahle D., Nadanasabesan N., Dias J., Valente L.M.P., Sørensen M., Kiron V. (2020). Approaches to Improve Utilization of Nannochloropsis Oceanica in Plant-Based Feeds for Atlantic Salmon. Aquaculture.

[110] Grammes F., Reveco F.E., Romarheim O.H., Landsverk T., Mydland L.T., Øverland M. (2013). *Candida Utilis* and *Chlorella Vulgaris* Counteract Intestinal Inflammation in Atlantic Salmon (*Salmo salar* L.). PLoS ONE.

[111] Agboola J.O., Øverland M., Skrede A., Hansen J.Ø. (2021). Yeast as Major Protein-Rich Ingredient in Aquafeeds: A Review of the Implications for Aquaculture Production. Rev. Aquac..

[112] Øverland M., Karlsson A., Mydland L.T., Romarheim O.H., Skrede A. (2013). Evaluation of *Candida utilis*, *Kluyveromyces marxianus* and *Saccharomyces cerevisiae* Yeasts as Protein Sources in Diets for Atlantic Salmon (*Salmo salar*). Aquaculture.

[113] Naylor R.L., Folke C., Kofinas G.P., Chapin F.S. (2009). Managing Food Production Systems for Resilience. Principles of Ecosystem Stewardship: Resilience-Based Natural Resource Management in a Changing World.

